# E-Learning for Medical Education in Sub-Saharan Africa and Low-Resource Settings: Viewpoint

**DOI:** 10.2196/12449

**Published:** 2019-01-09

**Authors:** Sandra Barteit, Albrecht Jahn, Sekelani S Banda, Till Bärnighausen, Annel Bowa, Geoffrey Chileshe, Dorota Guzek, Margarida Mendes Jorge, Sigrid Lüders, Gregory Malunga, Florian Neuhann

**Affiliations:** 1 Heidelberg Institute of Global Health Heidelberg Germany; 2 Ministry of Health Lusaka Zambia; 3 Department of Global Health and Population Harvard T.H. Chan School of Public Health Boston, MA United States; 4 Africa Health Research Institute (AHRI) KwaZulu-Natal South Africa; 5 Chainama College of Health Sciences Lusaka Zambia; 6 SolidarMed Lusaka Zambia

**Keywords:** medical e-learning, technology-enhanced learning, blended learning, health workers, health system strengthening, universal health coverage, medical education, mHealth, eHealth, developing countries, sub-Saharan Africa, low-resource countries

## Abstract

E-learning has been heralded as a revolutionary force for medical education, especially for low-resource countries still suffering from a dire lack of health care workers. However, despite over two decades of e-learning endeavors and interventions across sub-Saharan Africa and other low- and middle-income countries, e-learning for medical education has not gained momentum and continues to fall short of the anticipated revolution. Many e-learning interventions have been cul-de-sac pilots that have not been scaled up but rather terminated after the pilot phase. This is usually a result of not adopting a system-wide approach, which leads to insufficient scope of training, insufficient technological maintenance and user support, unattainably high expectations, and unrealistic financial planning. Thus, a multitude of e-learning evaluations have failed to provide scientifically sound evidence of the effectiveness of e-learning for medical education in low-resource countries. Instead, it appears that technological development has overwhelmed rather than revolutionized medical education. The question of how to push e-learning into a higher gear in low-resource countries persists. Provision of e-learning as a technology is insufficient. E-learning needs to be vigorously and sustainably integrated into the local educational setting and aligned with national strategies and other national endeavors and interventions. Adhering to a standardized framework for the implementation and evaluation of e-learning endeavors is key, especially to bridge the gap in robust evidence that should also guide e-learning implementations. The primary objective of e-learning for medical education is to strengthen the health system in order to serve the population’s health care needs and expectations. Currently, medical e-learning does not measure up to its potential or do justice to medical students in low-resource countries. Technology may help unfold the potential of e-learning, but an all-encompassing change is needed. This can only be achieved through a joint effort that follows a systematic and standardized framework, especially for implementation and evaluation.

## Introduction

The effect of electronic learning (e-learning) is likely to be revolutionary, although how precisely it will revamp professional education remains unknown.

This statement is part of a vision and strategy by Frenk et al (2010) for a commission on education and health workers for the 21st century [1]. As of 2018, the e-learning revolution in low-resource countries has not yet taken off, and the potential of e-learning in supporting the advancement of health training is still unknown.

What are the barriers that currently restrict the potential of e-learning for medical training in low-resource countries? How can e-learning address the continuing crisis in human resources for health? It is clear that progress is profoundly needed, considering that low-income countries are faced with fragile health systems and dramatically insufficient numbers of health workers. Of the 49 nations in sub-Saharan Africa with approximately one billion people, only about 6,000 medical doctors graduate per year as compared to Western Europe with a population of 200 million (one-fifth of sub-Saharan Africa), where the number of doctors that graduate per year (42,000) is seven times that in sub-Saharan Africa [1]. Low graduation numbers in sub-Saharan Africa reflect the small number of medical schools (134) in comparison to Western Europe (almost 300) [1]. As such, sub-Saharan Africa has an urgent need to scale up existing educational infrastructure to meet the large and increasing number of young people striving for education and to address the dire need for healthcare in their population by educating higher numbers of qualified health personnel. The current educational infrastructure (in particular, medical education), practical training for health workers, and continuous medical education are inadequate in quantity and quality. Institutions for medical education such as colleges of nursing, allied health sciences, and medical schools are faced with a limited capacity to enroll students and inadequate numbers of medical educators and are also affected by a lack of infrastructure such as an insufficient number of classrooms, lecture halls, or dorms.

Technology, on the other hand, is developing by leaps and bounds. Personal computers were introduced for the general public in the 1970s, and throughout the recent decades, technological advancement has been astonishingly rapid. Today, computers are widely available in many forms: tablets, mobile phones, laptops, and virtual reality headsets. These technologies change educational approaches and can potentially improve health care delivery. However, global digital resources are unequally distributed, causing a so-called “digital divide.” To empower low-income countries to fast-track the lengthy early development stages that high-income countries experience [1], narrowing of the digital gap by increasing the development of and access to technological resources is required. One fast-track technological advance is exemplified by the rapid and extensive coverage of mobile phones in sub-Saharan Africa [2]. Overall, sub-Saharan countries have progressed significantly in the past few years in mobile connectivity and internet access. This progress provides a fertile setting for medical e-learning; its advantages include flexible learning, time efficiency, potential lower costs (due to a reduced need for printed learning materials such as books, and easily updated e-materials), standardization of course content, distance delivery, and scalability. E-learning for medical education might provide these countries a chance to increase the numbers of trained health workers in low-income countries while maintaining or potentially improving the quality of education with the support of self-directed learning and thus decrease the workload of current health workers engaged in health education.

## Pilotitis: How to Overcome Pilots

Published literature has documented a multitude of e-learning interventions for medical education and electronic health (eHealth) in sub-Saharan Africa. In fact, there have been so many eHealth projects, that in 2012, Uganda issued a directive to halt all national mobile health (mHealth) initiatives and stopped pilots that used mobile and wireless devices for health across the country [3] despite the heralded revolution of health technology and its promised potential. Uganda reacted to the perceived chaos surrounding the pilots and paused to develop standards for technical specifications and align projects with Uganda’s national health strategy. Uganda is indicative of the general trend in sub-Saharan Africa in terms of e-learning for medical education using a myriad of approaches. E-learning interventions tend to be isolated or running in parallel in a single country, which evokes an impression of urgency, resulting in imprudent implementation based on a strategy of “anything is better than nothing.” The recent Global Observatory for eHealth of the World Health Organization [4] found that the overall number of pilots are decreasing and the number of implemented large-scaled projects are increasing; however, the recent report of the Broadband Commission for Sustainable Development states that there is still no national coordination of digital health solutions in low- and middle-income countries, leading to a “fragmented ecosystem” [5]. In addition, many e-learning interventions do not take a system-wide approach [6], leaving out important aspects in the e-learning design plan. At a later stage, such exclusions evolve to major barriers of technology adoption and lead to frustrations and failure of implementing the intervention beyond the pilot phase, such as insufficient training of all stakeholders (students, teachers, and administration), insufficient maintenance and technological user support, unattainably high expectations, and unrealistic financial planning [7]. As a result, many of these technology-enhanced interventions and projects never progress past the pilot stage [5]; they end before scale-up—a phenomenon named *pilotitis* [6]. Clearly, pilots are important as a small-scale testing phase before investing in a large-scale deployment, but the pilot requires clear-defined objectives paired with monitoring and evaluation [7]. Sustainable implementation leading to scale-up is crucial for medical e-learning to realize its full potential.

## Structured and Sustainable Implementation

To be successful, e-learning interventions need to follow a systemic approach in a given educational environment and more specifically consider the definition of scope, objectives and target group, availability of a corresponding curriculum, active involvement of teachers and administrators, sufficient information technology (IT) support, adequate IT infrastructure, and clear political and institutional support.

A standard evaluation framework can provide insight into adapting medical e-learning pilots to local conditions and needs. Interventions should be interwoven as tightly as possible within the local educational infrastructure [8]. At the infrastructural core are individuals who are committed learners; teachers; and administrative and support staff such as the head of the department, teaching coordinators, IT support, and network administrators. The curriculum provides guidance at the individual level, which is crucial for e-learning interventions for medical education, especially for integration of e-learning into the curriculum. However, as the commission on education and health professionals for the 21st century has noted, “professional education has not kept pace...largely because of fragmented, outdated, and static curricula that produce ill-equipped graduates,” as “local educational standards are all too often driven by the desire to fit into frameworks that are in place elsewhere” [1]. Thus, the introduction of e-learning can be a chance to overhaul current curricula to consider and integrate scientific advancements, learner-centered models that are competence oriented, and technology-enhanced teaching and learning methods.

The internet and other technologies have fostered the growth of knowledge that is generally available free of cost for anyone with the technological means of access. Thus, nowadays, knowledge is accessible like never before. Just a few decades ago, when most current medical curricula were developed, memorization of facts was often considered paramount. Today, the skill to locate necessary information has become more critical as part of synthesis, analysis, and decision-making processes [1]. With the advent of new learning technologies and changes in knowledge handling, educational institutions in low-income countries should use this opportunity to update their curricula [1]. The incorporation of e-learning in the curriculum would confirm its role as a significant educational device to foster progress, rather than the current perception of e-learning as a “technological toy.” In low-income countries facing insufficient numbers of medical teachers and in need of making fast improvements, e-learning could be an essential step to relieve the capacity overload of the small number of medical teachers by embracing new technological approaches in order to acquire knowledge and skills. For example, instead of a teacher-centered approach, a blended learning approach could be put in place wherein students learn with self-directed e-learning materials such as interactive quizzes, videos, and literature to deepen the understanding and cover a range of topics or modules. This e-learning component could be blended with face-to-face exchanges and thus decrease the time invested by medical teachers by substituting study discussion groups or a flipped classroom, wherein the medical teacher facilitates a discussion and a question-and-answer session. This would remove the need and time invested to prepare full lectures, but still target students’ needs with question-and-answer sessions.

The curriculum change is a crucial element for successful e-learning integration, but it is only one piece of the “puzzle.“ The successful implementation and sound evaluation must follow a multilevel approach that incorporates the individual learner, the learning environment, the context of the e-learning implementation, the technological environment, and the pedagogics involved in the e-learning implementation [9].

Before starting an e-learning implementation, the objectives and expected outcomes of the intervention should be clearly defined. On an individual level, a needs assessment can provide valuable insights for implementation regarding the content, e-learning design, and technological equipment. This would answer questions about what content the learners expect and what content can be offered. Materials should be targeted at the learner, consider cultural context, and align with national health strategies and guidelines. To develop learning materials, the input and active involvement of medical staff is needed, which constitute a bottleneck because e-learning often aims to bridge the lack of medical teachers. Hence, a strong commitment to a substantial initial investment in medical e-learning is required from medical teachers, institutional administration, and governing bodies [8]. Without convincing and contextual contents, medical e-learning remains a skeleton without meaningful outcomes.

Another important aspect is how the medical e-learning content should be designed and how learners and teachers can best use the e-learning materials. For content design, the users’ needs should be evaluated and a pedagogical e-learning strategy according to the curriculum should be established. Current learning-management systems such as the popular and open-source software Moodle [10] already offer a pedagogical framework that can be assembled to local needs and standards. The content should follow a standard model such as SCORM (shareable content object reference model) [11] to ensure compatibility with other software platforms and enable potential sharing of content. Pros and cons of what technology is best to employ should be carefully balanced within the given setting, especially with regard to the users (learners and teachers), support services (information and communication technology staff), and technological sustainability including long-term maintenance options and technological utility in the given low-resource setting. Are the users able to use the device without too much effort? Can the technological device be repaired in cases of damage within the country? Is the technological device fit for the environment? It may be necessary to provide technical devices to learners and teachers to ensure equal access to medical e-learning. Is access mainly online or offline? Should the e-learning platform be provided via a local server or a rented service? Who can maintain and update the technological infrastructure? The answers to these questions should guide the selection of technical devices and services. Financial planning also needs to consider reoccurring costs for technical equipment, ICT training domain-name registration, and data safety and confidentiality.

One remedy against *pilotitis* could be the technology itself. For example, an online database similar to a clinical trial registry could be established, by which eHealth and e-learning interventions for sub-Saharan Africa (or even globally) are centrally registered. This database could provide transparency to the current black box of eHealth and medical e-learning interventions. Potentially, existing institutions and efforts, for example, the Broadband Commission for Sustainable Development or the World Health Organization as part of the Global Observatory for eHealth, could provide frameworks and potentially, resources to host, maintain, and develop such an eHealth and medical e-learning database. This would ease collaboration, planning, and priority setting on national and international levels. The most-effective interventions among ongoing interventions could then be implemented where they are needed the most. Parameters such as the type of technology used for the intervention as well as evaluation methods and measured outcomes could be integrated into the database, thus supporting the advancement of standard evaluation methods for medical e-learning. This database could be a point of entry for new stakeholders as well as inform policymakers and regulatory bodies. National policies should support medical e-learning interventions and provide the necessary framework for structured growth of e-learning initiatives.

## The Alpha and Omega of E-Learning: Evaluation

Many published medical e-learning evaluations have low-quality scientific standards [12] and often report their results in a narrative manner [12-14] without following a reporting standard for either qualitative or quantitative evaluation. The majority of e-learning interventions rely on self-designed evaluation designs that are rarely validated. Subjective evidence generally focuses solely on the individual learners, such as learner satisfaction or other user-perceived parameters (eg, learner opinion). Studies that only incorporate the individual learner in their evaluation are restricted in their insight into the intervention outcome. Consequently, a tailored adaptation to the learners’ actual needs and requirements is difficult to achieve. Another limitation of this approach is that evidence from published medical e-learning studies cannot be used for comparison to other e-learning studies [12-14] and lessons that may have been learned from comparison are lost. Publications have tackled e-learning evaluation concepts and models [15-17] by emphasizing the need to comprehensively evaluate all levels of an e-learning intervention—from the learner to the institutional and governmental contexts. However, the heterogeneity of studies and the prominence of subjective evidence has led to a continued lack of sound scientific evidence on the effect of e-learning in medical education in low-resource countries. Therefore, a comprehensive evaluation of medical e-learning interventions is needed to generate causal evidence. The reasons for the predominance of trial and error pilots and the prevalence of “homemade” e-learning evaluations remain unclear. Resources to guide implementation and evaluation of e-learning interventions are plenty. Although fully randomized controlled trials might be difficult to conduct in regulated systems such as medical education, other causal research designs can and should be employed. Many methods like the objective structured clinical examinations or standardized multiple-choice tests are available for skill and knowledge testing. Prevalent models in e-learning for questionnaires include the technology acceptance models (TAM1 and TAM2) for assessing technology acceptance, the unified theory of acceptance and use, and the system usability scale for usability.

The CONSORT (Consolidated Standards of Reporting Trials) statement and NOS-E (Newcastle-Ottawa Scale for Education) include standards for reporting and assessing study quality. The primary objective for most interventions is to employ e-learning to strengthen medical education and provide good-quality education. Therefore, the implementation and evolution of e-learning should target these objectives and produce evidence that allows for causal conclusions.

## Future Work

In our opinion, a fundamental requirement of e-learning for medical education should be systematic, especially with regard to planning, implementation, and evaluation. Adherence to a general and standard implementation and evaluation framework that can be further refined for the local setting and employment of more causal research designs could improve sustainability and the quality of evidence. However, e-learning for medical education in low-resource countries still requires evidence to prove equivalence or even superiority to analogue and traditional educational approaches. A clear strategy for adapted implementation and integration as well as the understanding that e-learning is not a cure for all, but rather a valuable tool requiring a strong commitment and significant upfront investment with continued support, is necessary. In particular, a definition of new roles and potentially, the creation of new staff positions are needed. To move forward with this new structure, political will is essential for establishing national and regional strategies. Thus, international partnerships may provide a strong foundation of resources for initial setup. Alongside the digital educational infrastructure with e-learning for medical education in low-resource countries, health systems can be structurally and substantially strengthened with eHealth initiatives.

## Conclusions

E-learning for medical education in low-resource countries is in a disorganized state, with many pilot projects that are not scaled up. Therefore, the potential of e-learning, especially for medical education, remains underutilized. Making technology available is not sufficient nor does it do justice to the many potential general and medical students who need quality education in sub-Saharan Africa. We are not suggesting that e-learning is the magic bullet to solve existing problems with medical training in resource-limited settings with a lack of health workers and qualified medical educators. However, we see e-learning as an important and potent component with a potential that remains mostly underexploited to date in these settings. Therefore, we propose a concerted effort to unfold and enhance the effectiveness of e-learning as an educational tool to increase the quantity and quality of medical education programs. We suggest (1) a topic-specific database that registers all e-learning and eHealth interventions similar to clinical trial registers, (2) a standardized and widely employed framework for the evaluation of e-learning programs, and (3) structured programs that incorporate e-learning within and between medical teaching institutions and accreditation bodies. E-learning for medical education is not a self-runner: It requires significant upfront investment in content, training, and technology as well as the acknowledgment of recurring costs that will potentially be paid off only at a later stage [8]. For low-resource countries struggling to increase the number of health workers, medical e-learning may be able to accelerate progress by skipping many previous developmental steps taken by high-income countries in the past. Available technology, including software and hardware, has the potential to fundamentally change and shape health systems, the quality of health education, and subsequently, the quality and quantity of health care delivery and access. Stakeholders need to focus on formulating and promoting standard frameworks for implementation and evaluation [16]. Health is about people and so is medical e-learning. We need to emphasize that education is at the core of strengthening health systems as education enhances the performance of health systems, so that they can meet the needs of patients and populations equitably and efficiently.

## Abbreviations

CONSORT: consolidated standards of reporting trials
eHealth: electronic Health
IT: information technology
mHealth: mobile Health
NOS-E: Newcastle-Ottawa Scale for Education
TAM: technology acceptance model
